# New Therapy for Spinal Cord Injury: Autologous Genetically-Enriched Leucoconcentrate Integrated with Epidural Electrical Stimulation

**DOI:** 10.3390/cells11010144

**Published:** 2022-01-02

**Authors:** Rustem Islamov, Farid Bashirov, Andrei Izmailov, Filip Fadeev, Vage Markosyan, Mikhail Sokolov, Maksim Shmarov, Denis Logunov, Boris Naroditsky, Igor Lavrov

**Affiliations:** 1 Department of Medical biology and Genetics, Kazan State Medical University, 420012 Kazan, Russia; faridbashirov@yandex.ru (F.B.); andrei.izmaylov@kazangmu.ru (A.I.); philip.fadeyev@gmail.com (F.F.); vage.markosyan@gmail.com (V.M.); supermihon@yandex.ru (M.S.); 2 The National Research Center for Epidemiology and Microbiology Named after Honorary Academician N.F. Gamaleya of the Ministry of Health of the Russian Federation, 123098 Moscow, Russia; mmshmarov@gmail.com (M.S.); ldenisy@gmail.com (D.L.); bsnar1941@yahoo.com (B.N.); 3 Center for Neurobiology and Brain Restoration, Skolkovo Institute of Science and Technology, 121205 Moscow, Russia; 4 Institute of Fundamental Medicine and Biology, Kazan Federal University, 420008 Kazan, Russia; 5 Department of Neurology, Mayo Clinic, Rochester, MN 55905, USA

**Keywords:** autologous genetically-enriched leucoconcentrate, chimeric adenoviral vector, vascular endothelial growth factor, glial cell line-derived neurotrophic factor, neural cell adhesion molecule, spinal cord contusion injury, epidural electrical stimulation, mini-pigs

## Abstract

The contemporary strategy for spinal cord injury (SCI) therapy aims to combine multiple approaches to control pathogenic mechanisms of neurodegeneration and stimulate neuroregeneration. In this study, a novel regenerative approach using an autologous leucoconcentrate enriched with transgenes encoding vascular endothelial growth factor (VEGF), glial cell line-derived neurotrophic factor (GDNF), and neural cell adhesion molecule (NCAM) combined with supra- and sub-lesional epidural electrical stimulation (EES) was tested on mini-pigs similar in morpho-physiological scale to humans. The complex analysis of the spinal cord recovery after a moderate contusion injury in treated mini-pigs compared to control animals revealed: better performance in behavioural and joint kinematics, restoration of electromyography characteristics, and improvement in selected immunohistology features related to cell survivability, synaptic protein expression, and glial reorganization above and below the injury. These results for the first time demonstrate the positive effect of intravenous infusion of autologous genetically-enriched leucoconcentrate producing recombinant molecules stimulating neuroregeneration combined with neuromodulation by translesional multisite EES on the restoration of the post-traumatic spinal cord in mini-pigs and suggest the high translational potential of this novel regenerative therapy for SCI patients.

## 1. Introduction

Currently proposed therapies for the spinal cord injury (SCI) include a combination of different therapies such as pharmacological treatment, neuromodulation, bioengineering, biotechnology strategies, and other approaches. The main pharmacological therapies aim to control neuroinflammation, ischaemia, apoptosis, excitotoxicity, myelin preservation, astrogliosis, and other mechanisms involved in the acute and chronic phases of SCI [1]. Neuromodulation with electrical stimulation or neurochemical agents targets and activates proprioceptive feedback spinal circuits to respond to supraspinal signals and facilitate the formation of a translesional spinal neural network, thereby integrating interrupted SCI circuits. Innovative bioengineering strategies after SCI have the potential to facilitate the restoration of upper or lower limb movements [2]. Functional electrical stimulation of the peripheral nerves and skeletal muscles has been introduced to assist with recovery after injury, focusing on direct muscle activation. Spinal cord stimulation has further demonstrated significant effects on the restoration of volitional motor control after complete SCI [3,4], providing great promise for patients with total loss of all motor and sensory function below the level of injury. Recently, Bonizzato and Martinez [5] introduced a neuroprosthetic device for electrical stimulation at motor cortex level for cortical locomotor control after SCI in a rat model. Accumulating evidence suggests that the effect of both invasive (epidural) and non-invasive (transcutaneous) stimulation is mediated through the activation of the dorsal roots and facilitates the delivery of sensory information to the spinal cord [6,7,8,9]. A combination of spinal cord stimulation with intensive rehabilitation has so far demonstrated the optimal restoration potential for SCI patients [4]. The development of new biotechnologies offers a wide range of products and novel techniques including biodegradable scaffolds, cell transplantation, and recombinant therapeutic gene delivery, which can be applied in different combinations.

Cell and gene therapies have been extensively used in pre-clinical investigations and recent clinical trials [10]. Neurotransplantation of neural stem cells [11], Schwann cells [12], olfactory ensheathing cells [13], bone marrow-derived mesenchymal stem cells [14], or umbilical cord blood-derived mononuclear cells [15] aims to provide neuroprotection and stimulate neuroregeneration. Delivery of recombinant therapeutic genes encoding biologically active molecules, such as neurotrophic (BDNF, GDNF, NGF) and growth factors (VEGF, IGF-1, FGF-2), cell adhesion molecules (NCAM, L1), as well as anti-inflammatory (IL-10) and anti-apoptotic (Bcl-2) molecules for the activation or inhibition of the certain cellular reactions at the lesion can modulate neuroplasticity in the post-traumatic spinal cord [16]. Particularly, the combination of cell and gene therapies, i.e., the transplantation of gene-engineered cells, provides a strong synergistic effects [17]. Combinatorial strategies using different cell types and different genes in recent years has led to a variety of new gene-cell constructs. For example, for SCI treatment, fibroblasts producing NGF [18] and NT-3 [19], Schwann cells producing NT-3 [20] and NCAM [21], oligodendrocyte precursor cells producing CNTF [22], neural progenitor cells producing NT-3 [23], mesenchymal stem cells producing BDNF [24], olfactory ensheathing cells producing GDNF [25], and many other combinations have been developed. The severity and complexity of SCI requires a combination of multiple techniques to cover different mechanisms of injury [26]. The rationale for the combination of multiple therapies is based on possible synergistic effects of each therapy on a specific target, which altogether results in positive outcomes in post-traumatic spinal cord morpho-functional recovery.

Recently, we translated spinal cord epidural electrical stimulation (EES) to a porcine model and further demonstrated the efficacy of supra- and sublesional EES [27,28]. We also demonstrated the benefits of triple gene therapy after intrathecal injection of umbilical cord blood mononuclear cells (UCBC) simultaneously transduced with adenoviral vectors (Ad5) carrying the genes encoding for endothelial growth factor (VEGF), glial cell line-derived neurotrophic factor (GDNF), and neural cell adhesion molecule (NCAM) [29,30]. Comparative analysis of EES in combination with cell-mediated gene therapy both in rats [27] and mini-pigs [29] demonstrated the advantages of this approach in severe SCI.

**Figure 1:**
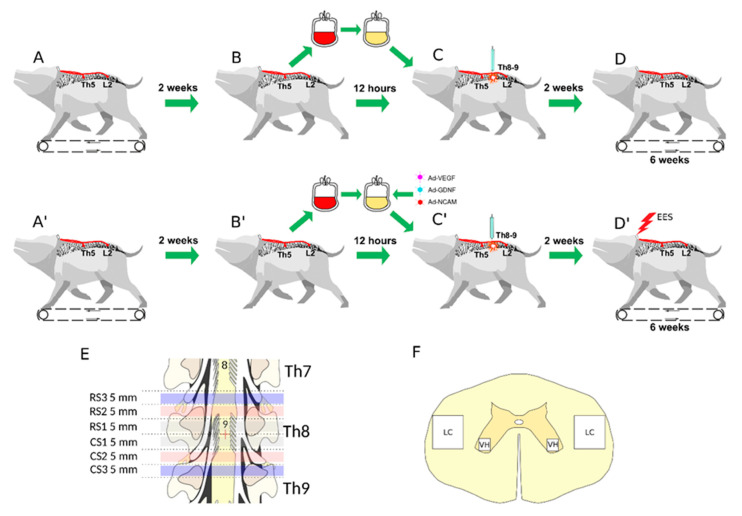
Study design. Upper panel shows control animals: (**A**) Implantation of stimulating electrodes at Th5 and L2 followed by recovery and training of the mini-pigs (*n* = 4) to walk on a treadmill. (**B**) Collection of 50 mL of venous blood and preparation of the leucoconcentrate. (**C**) Spinal cord contusion injury followed by intravenous infusion of the autologous leucoconcentrate 4 h after surgery. (**D**) Training on a treadmill every second day starting 2 weeks after injury. Behavioural and electrophysiological data were collected over the next 6 weeks. Middle panel shows treated animals: (**A’**) Implantation of stimulating electrodes at Th5 and L2 followed by recovery and training of the mini-pigs (*n* = 4) to walk on a treadmill. (**B’**) Collection of 50 mL of venous blood and preparation of the genetically-enriched leucoconcentrate. (**C’**) Spinal cord contusion injury followed by intravenous infusion of the autologous genetically-enriched leucoconcentrate 4 h after surgery. (**D’**) Epidural electrical stimulation (EES) every second day combined with training on a treadmill starting 2 weeks after injury. Behavioural and electrophysiological data were collected over the next 6 weeks. Low panel: (**E**) The spinal cords were harvested at 8 weeks after injury. The rostral (RS) and caudal (CS) segments of the spinal cord relative to the epicentre of injury (red cross) were divided into three segments: RS1, RS2, RS3 and CS1, CS2, CS3, correspondingly, for histological and molecular studies. (**F**) Spinal cord areas used for histological investigation: ventral horn (VH) and lateral column (LC).

In this study, for ex vivo gene therapy, we proposed using an autologous genetically-enriched leucoconcentrate producing recombinant VEGF, GDNF, and NCAM. UCBC used as cell carriers for ex vivo gene therapy have several limitations with regard to ethical controversies, the small amount of UCBC available from one donor, the restrictions associated with allotransplantation, and the difficulties of repeated infusions of UCBC from the same donor. The benefits of ex vivo gene therapy with leucoconcentrate as the gene delivery system, as proposed in this study, are based on availability (ease of obtaining cellular material), biosafety (lack of immune reactions and absence of recipient contact with viral particles), and the controlled level production of recombinant therapeutic molecules (known level of transduction and amount of transplanted gene-modified leucocytes). For effective transduction of peripheral blood leucocytes, we started to use a chimeric adenoviral vector (Ad5/35F) with modified fibres, which has a high affinity for cluster of differentiation 46 (CD46), which is expressed on all nucleated blood cells [31]. We also developed a technology for the preparation of autologous genetically-enriched leucoconcentrate in plastic blood containers, which excludes manipulation in vitro, as well as the usage of bioproducts of animal origin and antibiotics [32]. This study was designed to evaluate the therapeutic efficacy of multisite EES in combination with ex vivo gene therapy using autologous genetically-enriched leucoconcentrate for the temporary production of recombinant VEGF, GDNF, and NCAM in mini-pigs after contusion injury of the spinal cord (Figure 1).

## 2. Materials and Methods

### 2.1. Preparation of Autologous Genetically-Enriched Leucoconcentrate

In our previous investigations for the delivery adenoviral vectors (Ad5) carrying recombinant DNA encoding biologically active molecules for the stimulation of neuroregeneration in mouse, rat, and mini-pig models, we employed human UCBC. The disadvantages of this strategy include the complicated procedure of gene-modified UCBC preparation and hypothetical application of UCBC as a gene delivery system in medical practice in adults drove us to discover a new, simple, safe, and effective approach for recombinant DNA delivery that may be used as a combined therapy to enhance the efficacy of other methods. In the current study, EES was combined with personalised ex vivo gene therapy where autologous white blood cells (WBC) were used as a gene delivery system and as a bioreactor for the temporary production of the recombinant therapeutic molecules.

#### 2.1.1. Chimeric Ad5/35F Viral Vectors Construction

Recombinant replication-defective viral vectors carrying *vegf165*, *gdnf*, *ncam1*, and green fluorescent protein (*gfp*) genes were created based on the human adenovirus serotype 5 with fibres derived from adenovirus serotype 35 fibres (Ad5/35). Ad5/35 construction was based on the method described by Graham et al. [33]. Two plasmids were constructed for this. One of them (pAd5-F35-frt) contained the full Ad5 genome (delta E1/E3) with fibres of Ad35, ITRs, expression cassette with the CMV promoter, Flp-recombinase gene, SV40-polyA, and frt recombination site. The second (pGamal) contained ITRs, the encapsulation site of Ad5, the frt recombination site, and an expression cassette with the CMV promoter, gene of interest, and SV40-polyA. The nucleotide sequences encoding VEGF165 (Gene Bank NM_001171626.1), GDNF (Gene Bank NM_000514.4), and NCAM1 (Gene Bank NM_001076682.2) were obtained by chemical synthesis in ‘Evrogen’. Human and reporter genes were cloned into the pGamal shuttle plasmid vector. Recombination was then carried out for pGamal-VEGF, pGamal-GDNF, pGamal-NCAM1, and pGamal-GFP with pAd5-F35-frt plasmids in HEK293 cells. Recombinant Ads Ad5/35-VEGF165, Ad5/35-GDNF, Ad5/35-NCAM1, and Ad5/35-GFP were grown in HEK-293 cell culture and purified by exclusion chromatography. The titres of Ad5/35-VEGF165 (2.0 × 10^9^ PFU/mL), Ad5/35-GDNF (7.0 × 10^10^ PFU/mL), Ad5/35-NCAM1 (5.0 × 10^10^ PFU/mL), and Ad5/35-GFP (2.5 × 10^9^ PFU/mL) were determined by the plaque formation technique in HEK-293 cells.

#### 2.1.2. Blood Collection

Sixteen hours before surgery, 50 mL of blood was collected from the subclavian vein of each experimental animal into a plastic blood bag with 35 mL of anticoagulant-preservative solution (CPDA) under anaesthesia with an intramuscular injection of Zoletil 100 (Virbac Laboratoires, Carros, France; 10 mg/kg) and Xyla (Interchemie werken ‘De Adelaar B.V., Castenray, The Netherlands; 40 mg/kg). After blood collection, animals were re-infused with 100 mL of saline via the auricular vein and returned to housing area for recovery.

#### 2.1.3. Leucoconcentrate Preparation

The procedure included three steps, as described previously [32]. In brief: (1) 100 mL of 6% hydroxyethyl starch was added into the plastic bag with 100 mL of blood. Subsequently, the bag was centrifuged (DP-2065 R PLUS, Buenos Aires, Argentina) at 34× *g* for 10 min at 10 °C, and the erythrocytes were discarded; (2) the bag with contents was again centrifuged as above, and the supernatant was squeezed out into a new plastic blood bag using a manual plasma extractor (FK-01, Leadcore, Ekaterinburg, Russia); (3) for washing, 0.9% NaCl was added to the bag at a 1:9 ratio, and the bag with the mixture was centrifuged at 490× *g* for 10 min at 10 °C. The obtained supernatant was discarded from the bag using a manual plasma extractor and the leftover WBC in the bag were considered as the leucoconcentrate.

#### 2.1.4. Transduction of the Leucoconcentrate with Chimeric Ad5/35F Viral Vectors

Transduction of the leucoconcentrate was performed in the plastic bag with a multiplicity of infection (MOI) equal to 10 according to the WBC count in the leucoconcentrate. The obtained leucoconcentrate was immediately treated with the chimeric adenoviral vectors in an equal ratio of each vector including Ad5/35F-VEGF165 (1/3), Ad5/35F-GDNF (1/3), and Ad5/35F-NCAM1 (1/3) and placed on a multifunctional digital rocking shaker (DRS-12, ELMI SIA, Riga, Latvia) at room temperature. After transduction for 12 h, saline was added to the bag at a 1:9 ratio with the leucoconcentrate and the mixture was centrifuged at 290× *g* for 10 min at 10 °C. After this, the supernatant was squeezed out of the bag and the solution remaining in the bag was considered as the genetically-enriched leucoconcentrate carrying three transgenes, encoding VEGF165, GDNF, and NCAM1. For the in vitro study, the leucoconcentrate was also transduced with Ad5/35F-EGFP with MOI = 10.

#### 2.1.5. Complete Blood Count

A complete blood count (CBC) in naïve peripheral blood, in the obtained leucoconcentrate, and in leucoconcentrate transduced with adenoviral vectors carrying therapeutic genes was performed using a Sysmex XP-300 apparatus (Sysmex Corporation, Kobe, Japan).

### 2.2. Transgene Expression Analysis in Genetically-Enriched Leucoconcentrate In Vitro

#### 2.2.1. Flow Cytometric Analysis

To assess the number of EGFP-positive cells, a BD FACS Aria III (BD Biosciences, San Jose, CA, USA) was used. The samples of the leucoconcentrate transduced with Ad5/35-EGFP were seeded on 10-cm untreated culture dishes and incubated for 72 h in RPMI-1640 medium (PanEco, Moscow, Russia) supplemented with 10% foetal bovine serum (FBS; Biosera, Nuaille, France) and a mixture of 100 U/mL penicillin and 100 μg/mL streptomycin (Thermo Fisher Scientific, Waltham, MA, USA). The cells were cultured at 37 °C in a humidified incubator with 95% O_2_ and 5% CO_2_. Flow cytometry data are expressed as the percentage of EGFP-positive cells.

#### 2.2.2. RT-PCR Assay

mRNA levels of *vegf165, gdnf,* and *ncam1* transgenes in the genetically-enriched leucoconcentrate simultaneously transduced by a combination of three chimeric adenoviral vectors Ad5/35-VEGF165, Ad5/35-GDNF, and Ad5/35-NCAM1, were analysed by real-time polymerase chain reaction (RT-PCR). Total RNA was isolated from the genetically-enriched WBC harvested 72 h after incubation in vitro using an ExtractRNA kit (Eurogen) in accordance with the manufacturer’s instructions. cDNA synthesis was performed using random hexamers and an MMLV RT kit (Eurogen). Quantification of gene expression was carried out using a CFX96 thermal cycler (BioRad). The reaction mixture included qPCRmix-HS SYBR (Eurogen), cDNA samples, and specific primers for VEGF165, GDNF, and NCAM1 (Table 1). Each reaction was carried out with two technical repeats. The ΔΔCt (Livak) method was used to calculate the average relative target genes expression normalised to *gapdh* [34].

#### 2.2.3. ELISA

Supernatants obtained 72 h after incubation of the genetically-enriched leucoconcentrate simultaneously transduced by a combination of three chimeric adenoviral vectors Ad5/35-VEGF165, Ad5/35-GDNF, and Ad5/35-NCAM1 and naïve leucoconcentrate were used for enzyme-linked immunosorbent assay (ELISA). Naïve and genetically genetically-enriched WBC were incubated in RPMI-1640 medium (PanEco) containing 10% FBS at 37 °C under 5% CO_2_. After 72 h of incubation, conditioned culture medium was collected, centrifuged at 4 °C at 1000× *g* for 10 min and stored at −80 °C before use. The levels of soluble recombinant human VEGF, GDNF, and NCAM were estimated using ELISA kits for Vascular Endothelial Growth Factor A (VEGFA) (Cloud-Clone, SEA143Hu), Human GDNF DuoSet ELISA kit (R&D Systems, Cat # DY212), and Cluster Of Differentiation 56 (CD56) (Cloud-Clone, SEB225Hu) according to the manufacturer’s instructions. Optical density was measured on a BioRad xMark multifunctional microplate spectrophotometer (BioRad, Hercules, CA, USA) at a wavelength of 450 nm. The analysis procedure was performed in three biological and two technical repetitions.

### 2.3. Animals and Treatment

Miniature Vietnamese pot-bellied mature female pigs (25–30 kg) were obtained from the Kazan State Academy of Veterinary Medicine by N.E. Bauman (Kazan, Russia). Two weeks before investigation, animals were kept separately (one per housing area) with a 12 h light/dark regimen at a temperature of 24–25 °C with controlled air conditioning and properly organised access to food and water. The animal protocols were conducted according to the guidelines of the Declaration of Helsinki, and approved by the Kazan State Medical University Animal Care and Use Committee (approval No. 5 dated 26 May 2020). All experimental procedures were performed in accordance with the standards to minimise animal suffering and the size of experimental groups.

#### 2.3.1. Anaesthesia and Postoperative Care

Starting 12 h before surgery, animals for were limited in terms of access to water and food and one hour before were injected intramuscularly with the antibiotic Baytril 2.5% (Bayer, Vladimir, Russia) 0.2 mL/kg. Anaesthesia was induced by intramuscular administration of Zoletil 100 (Virbac Sante Animale, Carros, France) at a dose of 10 mg/kg and maintained using inhalation apparatus (Minor Vet Optima, Zoomed, Moscow, Russia) with isoflurane (Laboratorios Karizoo, S.A., Barcelona, Spain) as 2.0%–2.5% mixture with oxygen. Operating areas were shaved and cleaned with 10% solution of povidone-iodine (Hemofarm, Vrsac, Serbia), then twice with an alcohol solution for external application with chlorhexidine bigluconate 0.05% (LLC ‘Rosbio’, St. Petersburg, Russia) and dressed with sterile material. On the operating table, the body temperature of the animals was maintained at 38 °C. Post-operative care was performed to minimise animal suffering and to maintain a stable state of health and avoid post-operative complications. All animals received proper antibacterial and analgesic infusions and vitamin therapy as described previously [28]. Postoperative wound dressing and control of the implantation area was performed daily. Additionally, pain syndrome (vocalisation, depressed behaviour, and jitteriness), respiratory rate, body temperature, water and feed intake, and excretion of urine and faeces were assessed daily to the endpoint of the experiment.

#### 2.3.2. Electrode Implantation

In our previous study, we reported the procedure of stimulating electrodes implantation at the C5 and L2 spinal cord segments in mini-pigs with subsequent spinal contusion at the Th9 segment [29]. It was shown that sublesional EES at L2 activates central pattern generators and facilitates hind limb movements on a treadmill. Meanwhile, the supralesional EES at C5 did not affect locomotion in the front legs due to supraspinal control. In this study, electrodes were implanted two weeks before SCI at Th9 following the same protocol but with changing the supralesional stimulation site from C5 to Th5 segment with the idea that EES located closer to the injury may facilitate the newly growing axons through the injury, thereby facilitating the formation of a translesional network and promoting interactions between neural networks above and below the injury [35]. Animals were implanted with stimulating and reference Teflon-coated wire electrodes (AS632, Cooner Wire Company, Chatsworth, CA, USA). The thoracic stimulating electrode was fixed to the dura matter with a suture at the Th5 segment after a laminectomy of the Th5 vertebrae. The lumbar stimulating electrode was fixed to the dura matter with a suture at the L2 segment after a laminectomy of the L1 vertebrae. The reference electrodes were implanted intramuscularly into the m. erector spinae at the thoracic and lumbar regions. For connection to the electrophysiological equipment, implanted electrodes were connected to a 12-channel connector (Omnetics Connector Corporation, Minneapolis, MN, USA), which was placed under the skin on the withers site lateral to the midline.

#### 2.3.3. Training on the Treadmill

One week after electrode implantation, experimental mini-pigs were trained to walk on a treadmill by Torneo T-530 Olympia (Zhejiang kingdom sports Co., Ltd., Huzhou City, Zhejiang Province, China). The training was performed every second day for 30 min in the morning and 30 min in the evening. During training, mini-pigs were secured in a body weight support system so that the animals remained within the treadmill and the load on the hind limbs was from 5% to 20% of their weight. The treadmill belt speed was matched with hind limb stepping that was about 0.3–0.4 m/s.

#### 2.3.4. Spinal Cord Injury

A moderate contusion injury of the spinal cord was performed as described in our previous study [29], 14 days after implantation of the electrodes. After laminectomy at the Th8-Th9 vertebral level, the dura matter was exposed and a 50 g metal rod with a diameter of 9 mm of was centred. A single blow to the spinal cord with a metal rod falling from a height of 50 cm was performed. Hind limb skeletal muscle contraction and the appearance of a consistent hematoma at the site exposed to the metal rod confirmed the onset of SCI. Moderate contusion injury in mini pigs has demonstrated consistent morphological and behavioural changes across our previous studies [28,29,32,36].

#### 2.3.5. Autoinfusion of Genetically-Enriched Leucoconcentrate

Genetically-enriched leucoconcentrate was prepared 14 h before SCI, as described above. Four hours after SCI, mini-pigs in the therapeutic group received an intravenous infusion of 30 mL of the autologous genetically-enriched leucoconcentrate via the auricular vein.

#### 2.3.6. Epidural Electrical Stimulation

Supra- and sublesional EES (Th5 and L2 spinal levels) was performed in all animals from the therapeutic group during treadmill training from 2 to 8 weeks after SCI. For EES a Digitmer DS7A (Digitmer Ltd., Welwyn Garden, UK) was used. Stimulation parameters were configurated using LabChart data acquisition and analysis system (AD Instruments Inc., Colorado Springs, CO, USA). The current used for EES was adjusted to not cause any discomfort and was selected individually for each animal. EES was conducted every second day for 30 min in the morning session (supralesion site, at Th5) and in the evening session (sublesional site, at L2) with an interval of 6–8 h between both sessions. During the morning procedure, stimulation (7–15 mA, 20–25 Hz, pulse duration 0.2 ms) to facilitate the activity of neurons above the injury was performed at the Th5 level. In the evening session, stimulation at the L2 level was applied to activate central pattern generators to stimulate active hind limb movements. The parameters of EES (13–25 mA, 20–25 Hz, and pulse duration of 0.2 ms) were selected such that animals produced walking movements matched with the speed of the treadmill belt.

#### 2.3.7. Experimental Groups

In the current study, experimental mini-pigs were divided into three groups: intact group (I, *n* = 4), control group (C, *n* = 4), and therapeutic group (T, *n* = 4). Animals from the therapeutic and control groups after implantation of the stimulating electrodes were subjected to SCI. Subsequently, mini-pigs in the therapeutic group were infused with genetically-enriched autologous leucoconcentrate and from 2 to 8 weeks after SCI underwent EES during training on a treadmill. Control mini-pigs after SCI were infused with autologous leucoconcentrate and were trained from 2 to 8 weeks after SCI on a treadmill. Healthy (intact) mini-pigs were used to collect basic molecular, histological, electrophysiological, and behavioural data (see below) for comparative analysis of the efficacy of gene and electrotherapy for SCI (Figure 1).

### 2.4. Post-Traumatic Spinal cord Functional Recovery

#### 2.4.1. Porcine Thoracic Injury Behavioural Scale

Recovery of motor function was assessed with the Porcine Thoracic Injury Behaviour Scale (PTIBS) [37]. A week before SCI induction and 2, 4, and 8 weeks after neurotrauma, hind limb movement activity was characterised ranging from no hind limb movements (score 1) to normal walking (score 10). PTIBS scores equal to 1–3 corresponded to “hind limb dragging”, scores of 4–6 indicate various degrees of ‘stepping’, and scores of 7–10 indicate ‘walking’ ability. The score was estimated by two observers when the animal was placed in the centre of the arena (2.5 × 2.5 m) for the testing in a blinded manner with respect to the experimental group. PTIBS assessment was performed once a week and stepping performance was visually assessed and interrater agreement.

#### 2.4.2. Joint Kinematics

Hind limb joint kinematics were evaluated based on changes in angular degrees in hip, knee, and ankle joints while experimental mini-pigs were walking on a treadmill a week before SCI and 2, 4, and 8 weeks after contusion injury. Coloured marks were applied to projections of the ridge of the left iliac bone, trochanter of the femur, knee, ankle joint, and hoof. Afterwards, mini-pigs were placed on the treadmill with belt speed of 0.3–0.4 m/s as described above. Video recording of 5 walking cycles was performed with Canon PowerShot S5 IS camera (Canon, Tokyo, Japan). In the animals from the therapeutic group, the recording was performed in the absence of EES and during EES at the L2 level. Analysis of video recording was performed using Kinovea software 0.8.25 [38]. The joint movement range was calculated as a difference between the maximum and minimum joint angles when evaluating five walking cycles. R 3.4.4 (R Foundation for Statistical Computing, Vienna, Austria) was used to analyse and visualise the results of the joint kinematics study.

#### 2.4.3. Electrophysiological Study

The M-response evoked by electrical stimulation of the sciatic nerve were recorded in the soleus muscle of left hind limbs before SCI and 2, 4, and 8 weeks after contusion injury. Under deep anaesthesia, two needle stimulating electrodes (stainless steel, diameter 0.6 mm, length 50 mm) were introduced into the projection of the sciatic nerve (2 cm below the greater trochanter of the femur and 1 cm down from the femur). Muscle responses were recorded using needle electrodes (stainless steel, diameter 0.6 mm, length 50 mm), which were inserted into the soleus muscle at the site between the medial and lateral heads of gastrocnemius muscle. The recording needle electrodes were injected into the soleus muscle (described previously). Sciatic nerve stimulation parameters were configurated using a Digitimer DS7A (Digitimer Ltd., Welwyn Garden, UK). Single rectangular pulses with a frequency of 0.6 Hz, duration of 0.2 ms, and a current range of 4–72 mA were used. Evoked biopotentials were amplified (Biosignal amplifier, g.tecmedical engineering GmbH, Schieldberg, Austria) and analysed using the LabChart data acquisition and analysis system (AD Instruments Inc., Colorado Springs, CO, USA).

### 2.5. Post-Traumatic Spinal Cord Molecular and Cellular Changes

#### 2.5.1. Sample Collection

Animals from the control and therapeutic groups were culled 8 weeks after SCI and the spine was isolated along the epicentre of the injury from Th5 to Th10. The spinal cord was removed from the vertebral column and divided into rostral (RS) and caudal (CS) segments, each 15 mm long, relative to the epicentre of the injury. The RS and CS were further subdivided into three equal subsegments and processed in accordance with the investigation methods (Figure 1E).

#### 2.5.2. Immunofluorescence Staining

Free-floating cross sections of 20 μm thickness were prepared from the RS2 and CS2 segments (Figure 1E,F). Antibodies (Ab) to potassium-chloride cotransporter protein (KCC2), heat shock protein of 27 kDa (Hsp27), and a pro-apoptotic protein (caspase-3) were employed to evaluate the survivability of spinal cord cells. The functional recovery of neural cells was analysed with Abs against synaptophysin and postsynaptic density protein of 95 kDa (PSD95). Astrocytes were identified with an antibody to glial fibrillary acidic protein (GFAP), oligodendroglial cells with an antibody to oligodendrocyte transcription factor (Olig2), and microglial cells with an antibody to ionised calcium binding adaptor molecule 1 (Iba1). An antibody to neuronal specific tubulin (βIII-tubulin) was used to identify axons in the region of the lateral corticospinal tracts of RS1 and CS1 segments on the longitudinal 10 μm thickness slide mounted sections. The appropriate secondary antibodies were used to visualise the immune complexes (Table 2). Nuclear counterstaining was performed with DAPI (10 μg/mL in PBS, Sigma, Burlington, MA, USA) and sections were embedded in glycerol (GalenoPharm, Saint Petersburg, Russia). Digital images were taken with a luminescence microscope (Axioscope A1 (Carl Zeiss, Oberkochen, Germany) and Leica TCS SP5 MP (Leica Microsystems, Wetzlar, Germany)) using identical settings. The pattern of target protein expression was evaluated in the ventral horns of grey matter and in the lateral corticospinal tract region of the white matter with an area of 0.05 mm^2^ using ImageJ (NIH) software. The immunopositive area was calculated as the ratio of the number of positive pixels to all pixels in the analysed area. The number of positive pixels was calculated automatically using the ‘threshold’ option in ImageJ. The number of immunopositive cells expressing caspase-3 and Olig2 was counted with regard to nuclear counterstaining with DAPI. The level of Hsp27, synaptophysin, PSD95, KCC2, βIII-tubulin, GFAP, and Iba1 expression was evaluated as the immunopositive area and presented as percentages.

#### 2.5.3. Synaptic Gene Expression

Analysis of the excitatory cholinergic system (pre-synaptic gene [*chat*] and the post-synaptic gene [*chrm1*] and inhibitory GABAergic system (pre-synaptic gene [*gad67*] and post-synaptic gene [*gabra2*]) in the RS3 and CS3 segments was performed using RT-PCR. Total RNA was isolated from the spinal cord tissue using ExtractRNA (Eurogen) in accordance with the manufacturer’s instructions. cDNA synthesis was performed using random hexamers and MMLV RT kit (Eurogen). Quantification of gene expression was carried out using a CFX96 thermal cycler (BioRad). The reaction mixture included qPCRmix-HS SYBR (Eurogen), cDNA samples, and specific primers for the synaptic genes (Table 1). Expression levels of the *gapdh* gene were used for normalisation. Each reaction was carried out in two technical repeats. The ΔΔCt (Livak) method was used to calculate the average relative target gene expression normalised to *gapdh* [34].

### 2.6. Statistical Analysis

Statistical data analysis and visualisations were performed using R version 3.6.3 (R Foundation for Statistical Computing, Vienna, Austria). Sample distributions of quantitative values were visualised using box plots, and descriptive statistics are presented as: (median [1st quartile; 3rd quartile]). The Kruskal–Wallis test was used to compare morphometric results, immunofluorescent staining, and behavioural and electrophysiological measures between experimental groups. We used Dunn’s test as the post hoc method. The Wilcoxon signed-rank test was used to compare matched measurements of HSP positive relative areas between experimental groups. Differences were considered statistically significant where *p* < 0.05. The ∆∆Ct method was used to estimate relative differences in mRNA levels using *gapdh* gene transcripts as the reference for normalisation. The Tukey method used to calculate 95% confidence intervals.

## 3. Results

### 3.1. Complete Blood Count

From 50 mL of peripheral blood, the average volume of genetically-enriched leucoconcentrate was approximately 50 mL as well (Table 3). The total number of leucocytes, erythrocytes and platelets was significantly reduced by 1.6, 32.5 and 3.6 times in the genetically-enriched leucoconcentrate, respectively, when compared to peripheral blood. The lower number of WBC was due to the 2.4-fold decrease in lymphocytes.

### 3.2. Expression of the Recombinant Genes in the Genetically-Enriched Leucoconcentrate

**Figure 2:**
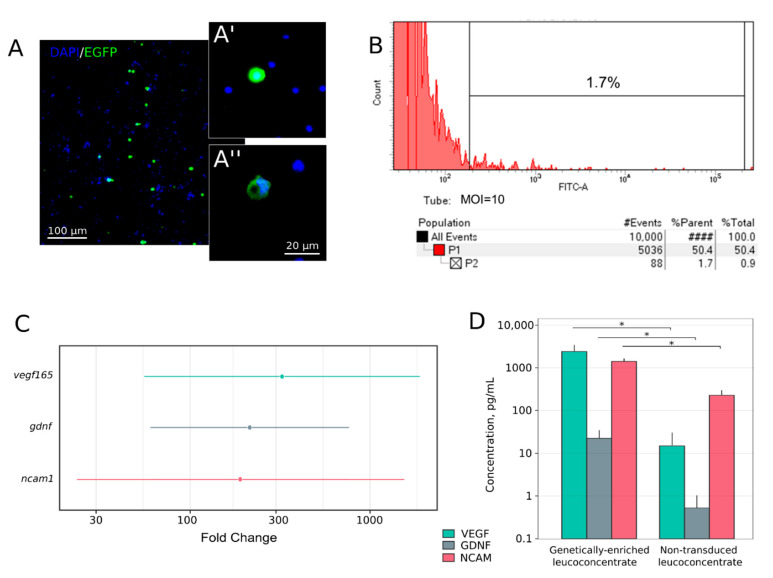
In vitro study of recombinant gene expression in the genetically-enriched leucoconcentrate 72 h after transduction of the leucoconcentrate with Ad5/35F-EGFP at MOI = 10. (**A**) Fluorescent microscopy demonstrating EGFP-positive cells with intensive green fluorescence. Nuclei are stained with Hoechst 33,342 (blue). Inserts demonstrate a lymphocyte (**A’**) and a monocyte (**A”**). (**B**) Flow cytometric analysis showing that 1.7% of WBC in the genetically-enriched leucoconcentrate produced green fluorescent protein. (**C**) Quantitative analysis of mRNA *vegf165*, *gdnf*, and *ncam1* levels by RT-PCR in genetically-enriched leucoconcentrate 72 h after in vitro incubation. The results are presented as average fold change and corresponding 95% confidence interval in the genetically-enriched leucoconcentrate relative to the expression level in the non-transduced leucoconcentrate. (**D**) ELISA analysis of recombinant VEGF, GDNF, and NCAM levels in the conditioned culture medium 72 h after incubation of the genetically-enriched and non-transduced leucoconcentrate. * *p* < 0.05.

Expression of the reporter *egfp* gene in the leucoconcentrate transduced with Ad5/35-EGFP was analysed using fluorescent microscopy and flow cytometry. Intensive green fluorescence was shown in WBCs identified as monocytes and lymphocytes (Figure 2A). Flow cytometric analysis revealed 1.7% of EGFP-positive cells in the leucoconcentrate transduced with Ad5/35F-EGFP at MOI = 10 (Figure 2B).

Expression *vegf165*, *gdnf*, and *ncam1* was investigated in samples of the genetically-enriched leucoconcentrate 72 h after in vitro incubation by RT-PCR, ELISA, and immunofluorescence methods. Analysis of the RT-PCR data demonstrated a significant increase in the mRNA level by 324.2-fold [55.5–1895.6] for *vegf165* (P = 0.0002), by 217.9-fold [61.1–777.0] for *gdnf* (P = 0.0001), and by 190.5-fold [23.4–1549.7] for *ncam1* (P = 0.0009) when compared to the mRNA level of the target genes in samples obtained from the non-transduced leucoconcentrate and incubated 72 h in vitro.

Production of recombinant VEGF, GDNF, and NCAM was evaluated in the conditioned culture medium 72 h after incubation of the genetically-enriched leucoconcentrate. ELISA revealed higher levels of VEGF by 163-fold (2620.9 pg/mL), GDNF by 43-fold (23.8 pg/mL), and NCAM by 2.2-fold (1523.9 pg/mL) compared to the non-transduced leucoconcentrate (15.68, 0.55, and 687.0 pg/mL, respectively).

### 3.3. Hind Limb Locomotor Function Recovery

**Figure 3:**
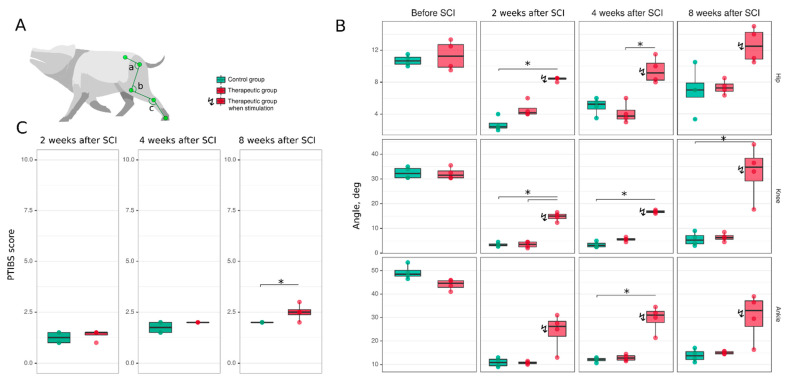
Hind limb locomotor function recovery in mini-pigs after spinal cord injury (SCI). (**A**) Video recording of the angle (degree) of movements in the hip (a), knee (b) and ankle (c) joints in mini-pigs while walking on a treadmill. The records were obtained in intact healthy mini-pigs one week before and at different time points after spinal cord injury. (**B**) Analysis of the kinematics in the hip, knee, and ankle joints in mini-pigs before and 2, 4, and 8 weeks after spinal cord injury in the control (green bars) and therapeutic (pink bars) groups. The mini-pigs from the therapeutic group were examined both without EES (as control animals) and when they were stimulated at L2. (**C**) Motor activity in control (green bars) and treated (pink bars) mini-pigs after spinal cord injury assessed with the PTIBS (Porcine Thoracic Injury Behavioural Scale). * *p* < 0.05.

Post-traumatic spinal cord functional recovery was evaluated based on hind limb locomotor activity. Mini-pigs from the control and therapeutic groups were tested a week before SCI and 2, 4, and 8 weeks after the spinal contusion injury (Figure 3). Hind limb movement activity was assessed with PTIBS and demonstrated severe suppression of motor activity at 2 and 4 weeks after SCI in both groups of animals. At 8 weeks, mini-pigs from the therapeutic group had higher PTIBS scores compared to control animals (*p* = 0.045) (Figure 3C). To evaluate the hind limb joint kinematics, the angles of flexion in the hip, knee, and ankle joints were measured while the experimental animals were walking on a treadmill. The animals from the therapeutic group were assessed with and without EES. At 2 weeks after SCI, the hind limb movements were decreased in both the control (hip—2.4 [2.2–2.9]; knee—3.2 [2.9–3.8]; ankle—10.8 [9.5–12.2]) and therapeutic (hip—4.2 [4.0–4.8]; knee—3.6 [2.5–4.5]; ankle—10.8 [10.4–11.1]) groups compared to data obtained from the control (hip—10.7 [10.2–11.1]; knee—32.2 [30.5–34.2]; ankle—48.5 [47.6–50.1]) and treated (hip—11.2 [9.9–12.7]; knee—31.5 [30.5–33.2]; ankle—44.6 [42.9–45.8]) animals before SCI (*p* < 0.05) (Figure 3A,B). Here and subsequently, the data are presented as: (median [1st quartile—3rd quartile]). During EES in treated mini-pigs 2 weeks after surgery, the angle of flexion was higher in the hip (8.5 [8.4–8.5], *p* = 0.1817), knee (15.0 [14.0–15.8], *p* = 0.0461), and ankle (26.2 [22.0–28.4], *p* = 0.0629) joints in comparison with the data collected without EES. At 4 weeks after SCI, there was no significant progress in the joint kinematics in both groups. The hip (5.2 [4.6–5.6]), knee (3.1 [2.5–4.0]), and ankle (12.2 [11.7–12.6]) joint movement range in control mini-pigs was similar with the angle of flexion in treated animals (hip—3.8 [3.4–4.5]; knee—5.6 [5.2–5.9]; ankle—12.8 [11.9–13.8]) group. In the therapeutic group during EES, the joint angles were higher in the hip (9.2 [8.2–10.4], *p* = 0.0237), knee (16.8 [16.4–17.1], *p* = 0.1899), and ankle (31.0 [27.8–32.6], *p* = 0.0694) joints and corresponded to the data collected after 2 weeks with EES. At 8 weeks, there was further progress in the hind limb joint movement range compared to the results at 4 weeks. An increase was seen in the angles of flexion in the joints in control animals (hip—7.0 [6.1–7.9]; knee—5.2 [3.8–7.1]; ankle—13.8 [12.1–15.5]) and in treated mini-pigs (hip—7.2 [6.8–7.8]; knee—6.3 [5.6–7.1]; ankle—15.0 [14.5–15.5]). In treated animals during EES, there was an increase in the movement of the hip (12.5 [10.9–14.2], *p* = 0.0528), knee (34.8 [29.2–38.4], *p* = 0.0620), and ankle (33.0 [26.2–37.1], *p* = 0.0783) joints. Importantly, at this time point, the flexion in the hip, knee, and ankle joints in the treated mini-pigs during EES was not significantly different from the corresponding values before SCI.

**Figure 4:**
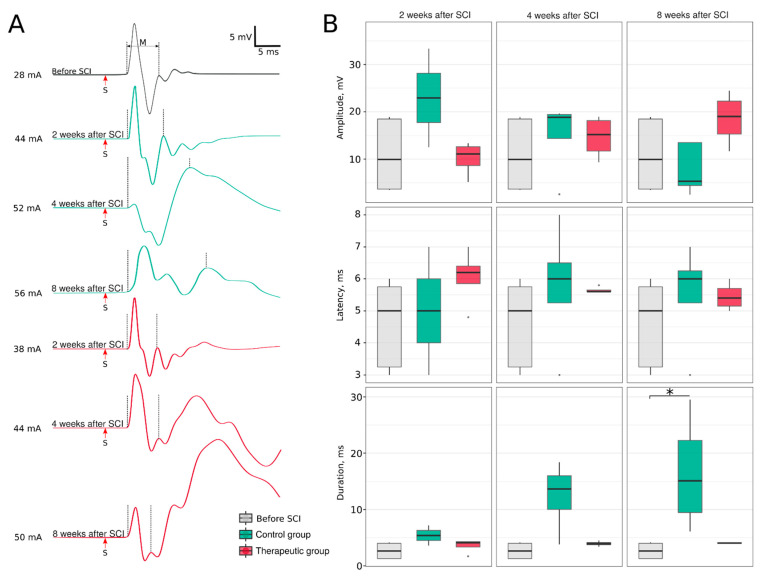
Electrophysiological evaluation of the soleus muscle induced by sciatic nerve stimulation. (**A**) Electromyography of the soleus muscle. (**B**) Parameters of the M-response, * *p* < 0.05.

Evaluation of the soleus muscle electromyography demonstrated an altered pattern of the M-response (Figure 4). In control mini-pigs, similar to our previous findings [28], the polyphasic M-response was associated with a gradual increase in response duration at 2 (54.0 [45.0–63.0]), 4 (136.5 [100.2–160.0], *p* = 0.0621) and 8 (151.0 [94.8–222.8], *p* = 0.0044) weeks, when compared to the M-response in intact animals (26.5 ([3.0–40.0]). The restoration of the M-response shape and duration at 2 (41.0 [33.5–43.0]), 4 (39.5 [37.8–41.2]) and 8 (40.5 [39.8–41.0]) weeks after SCI suggest the possible recovery of the motor units in treated animals. Amplitude and latency did not show any significant differences across experimental groups. An analysis of the average weight (g) of m. soleus at 8 weeks after SCI revealed a decrease in muscle weight in the control group (3.5 [3.5–5.5]) compared to the intact group (9.9 [9.3–11.3]) (*p* = 0.0006). In the treated mini-pigs, the weight of m. soleus was 6.0 [4.7–8.2] and was not different from the intact group.

### 3.4. Post-Traumatic Spinal Cord Molecular and Cellular Changes

#### 3.4.1. Immunofluorescence Study

Molecular and cellular changes in the post-traumatic spinal cord were investigated by immunofluorescence in the ventral horns of the rostral (RS2) and caudal (CS2) segments.

**Figure 5:**
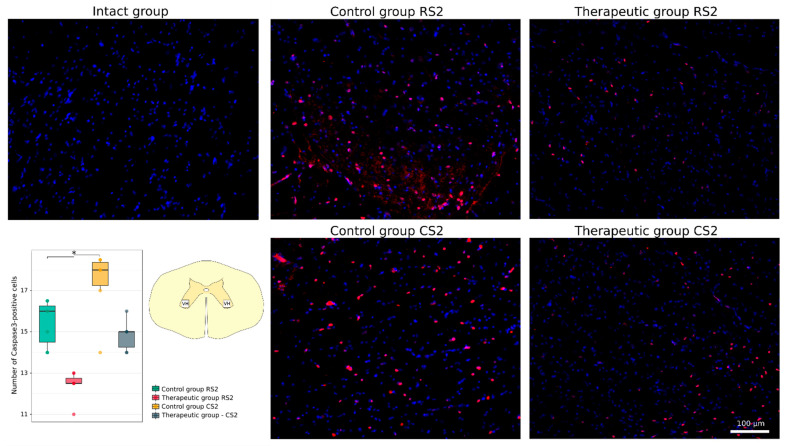
Immunofluorescence staining of the ventral horns of the spinal cords in the rostral (RS2) and caudal (CS2) segments 8 weeks after contusion injury with an antibody against a pro-apoptotic protein (caspase-3) (red). Nuclei were counterstained with DAPI (blue). Morphometric analysis demonstrates the number of caspase3-positive nuclei in the intact, control and therapeutic groups in the corresponding areas, * *p* < 0.05. The squares inserted in the schematic transverse spinal cord image demonstrates the areas used for immunofluorescence analysis, VH—ventral horn.

**Figure 6:**
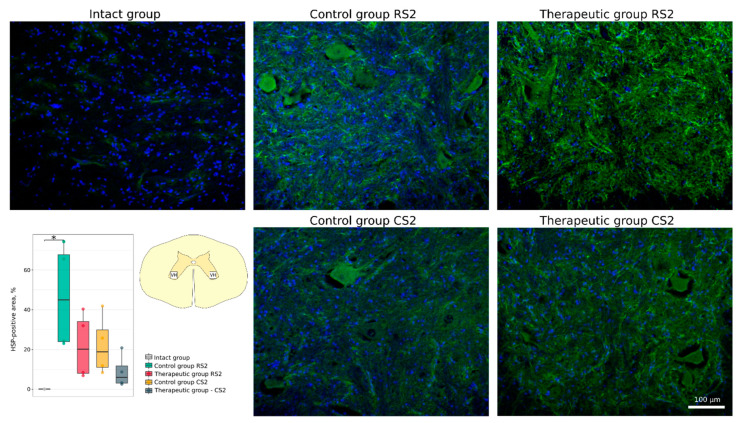
Immunofluorescence staining of the ventral horns of the spinal cords in the rostral (RS2) and caudal (CS2) segments 8 weeks after contusion injury with an antibody against heat shock protein 27 kDa (Hsp27) (green). Nuclei were counterstained with DAPI (blue). Morphometric analysis demonstrates the mean (%) of the Hsp27-positive area in the intact, control and therapeutic groups in the corresponding areas, * *p* < 0.05. The squares inserted in the schematic transverse spinal cord image demonstrates the areas used for immunofluorescence analysis, VH—ventral horn.

**Figure 7:**
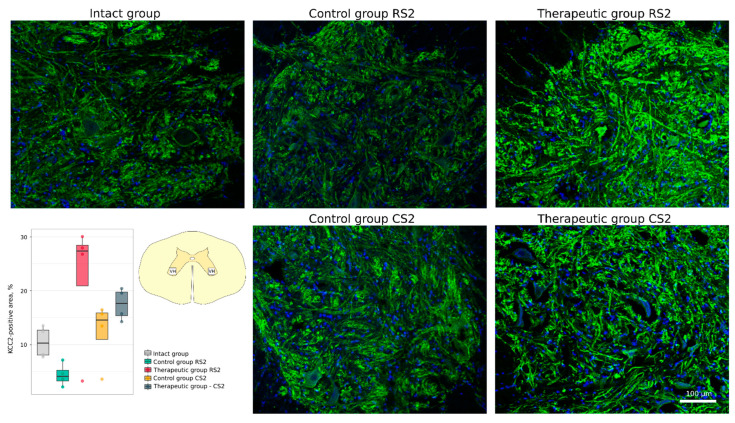
Immunofluorescence staining of the ventral horns of the spinal cords in the rostral (RS2) and caudal (CS2) segments 8 weeks after contusion injury with an antibody against potassium-chloride cotransporter protein 2 (KCC2) (green). Nuclei were counterstained with DAPI (blue). Morphometric analysis demonstrates the mean (%) of the KCC2-positive area in the intact, control and therapeutic groups in the corresponding areas. The squares inserted in the schematic transverse spinal cord image demonstrates the areas used for immunofluorescence analysis, VH—ventral horn.

The cellular stress response and apoptosis were investigated using Abs to heat shock protein 27 kDa (Hsp27), potassium-chloride cotransporter protein 2 (KCC2) and the pro-apoptotic protein caspase-3. The number of caspase-3-positive cells was higher in the rostral segment in the control group vs. the therapeutic group (16.0 [14.5–16.2] vs. 12.5 [12.5–12.8], *p* < 0.05) and non-significantly different in the caudal segments correspondingly (18.0 [17.2–18.4] vs. 15.0 [14.2–15.0]) (Figure 5). The Hsp27-positive area was increased in the rostral segment in the control group vs. the intact group (44.94 [24.05–67.70] vs. 0.12 [0.08–0.17], *p* < 0.05). Non-significant increase was found in the caudal segment in the control group (18.87 [11.05–29.84]) and in the rostral and caudal segments in the therapeutic group (20.23 [8.10–34.05] and 6.04 [3.14–11.80]) (Figure 6). Analysis of KCC2-positive area in the ventral horn demonstrated different patterns of KCC2 expression in the rostral segment (control 4.15 [3.29–5.28] vs. therapeutic 27.37 [20.92–28.48]) and in the caudal segment (control 14.58 [11.00–15.89] vs. therapeutic 17.64 [15.36–19.77] relative to the intact group 10.33 [8.10–12.72]) (Figure 7).

**Figure 8:**
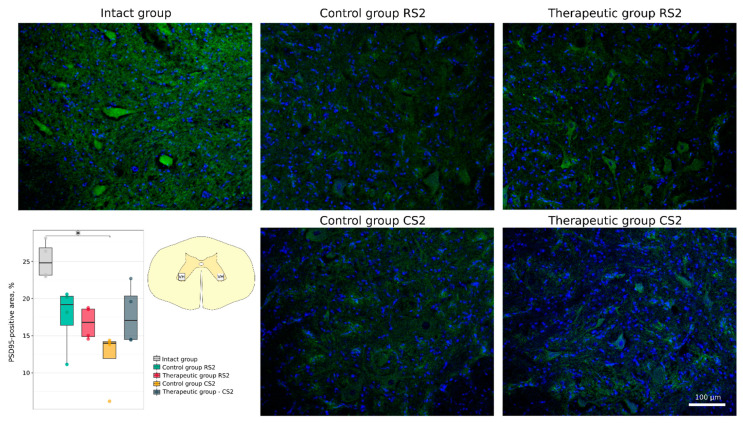
Immunofluorescence staining of the ventral horns of the spinal cords in the rostral (RS2) and caudal (CS2) segments 8 weeks after contusion injury with an antibody against postsynaptic density protein 95 kDa (green). Nuclei were counterstained with DAPI (blue). Morphometric analysis demonstrates the mean (%) of the PSD95-positive area in the intact, control and therapeutic groups in the corresponding areas, * *p* < 0.05. The squares inserted in the schematic transverse spinal cord image demonstrates the areas used for immunofluorescence analysis, VH—ventral horn.

**Figure 9:**
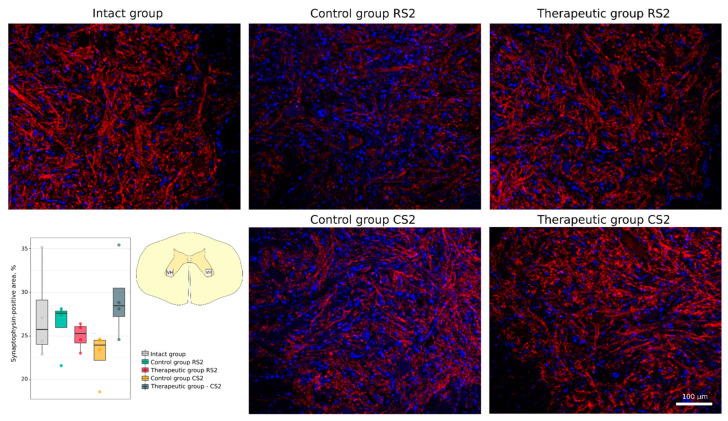
Immunofluorescence staining of the ventral horns of the spinal cords in the rostral (RS2) and caudal (CS2) segments 8 weeks after contusion injury with an antibody against the synaptic vesicle protein synaptophysin (red). Nuclei were counterstained with DAPI (blue). Morphometric analysis demonstrates the mean (%) of the synaptophysin-positive area in the intact, control and therapeutic groups in the corresponding areas. The squares inserted in the schematic transverse spinal cord image demonstrates the areas used for immunofluorescence analysis, VH—ventral horn.

**Figure 10:**
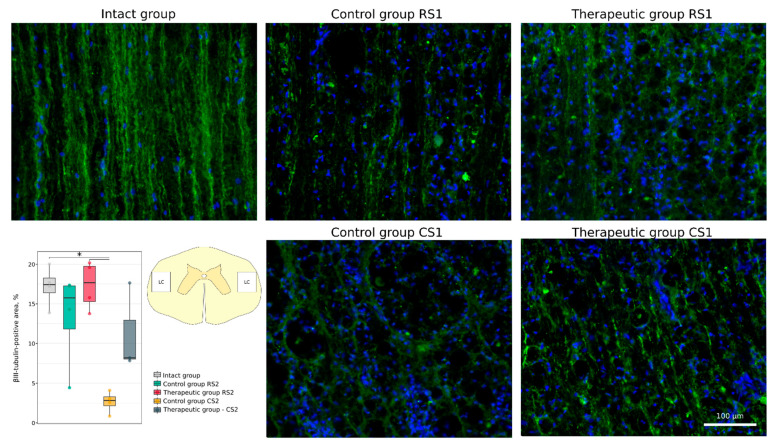
Immunofluorescence staining of the lateral columns of the spinal cords in the rostral (RS1) and caudal (CS1) segments 8 weeks after contusion injury with an antibody against βIII-tubulin (green). Nuclei were counterstained with DAPI (blue). Morphometric analysis demonstrates the mean (%) of the βIII-tubulin-positive area in the intact, control and therapeutic groups in the corresponding areas, * *p* < 0.05. The squares inserted in the schematic transverse spinal cord image demonstrates the areas used for immunofluorescence analysis, LC—lateral column.

Functional recovery of neurons and axonal growth through the epicentre were analysed using Abs to postsynaptic density protein 95 kDa (PSD95), to synaptophysin and to neuronal specific tubulin (βIII-tubulin). A significant decrease in PSD95-positive area was demonstrated in the caudal segment in the control group vs. the intact group (14.0 [11.9–14.2]) vs. 24.8 [23.2–26.8], *p* < 0.05). At the same time, expression of PSD95 was similar in the rostral control (19.2 [16.4–20.3]) and therapeutic (16.8 [14.9–18.6]) segments and in the caudal control (14.0 [11.9–14.2]) and therapeutic (17.0 [14.5–20.4]) segments (Figure 8). No significant difference was found in synaptophysin-positive area in the rostral segments in the control (27.6 [26.0–27.9]) and therapeutic (25.3 [24.2–26.1]) groups and in the caudal segments in the control (24.0 [22.2–24.5]) and therapeutic (28.5 [27.2–30.5]) groups relative to the intact group (25.8 [24.0–29.1]) (Figure 9). Immunostaining for βIII-tubulin was observed in the lateral column on both sides of the white matter in the rostral and caudal segments near the epicentre (RS1 and CS1). The βIII-tubulin-positive area was similar in the rostral segments of control (15.77 [11.83–17.26]) and treated (17.67 [15.27–19.72]) animals when compared with intact (17.43 [16.38–18.25]) mini-pigs. βIII-tubulin-positive area was significantly smaller in the caudal segment in the control group (2.81 [2.15–3.30]) (*p* < 0.05) and did not differ in the therapeutic group (8.21 [8.03–12.92]) when compared with the intact group (Figure 10).

**Figure 11:**
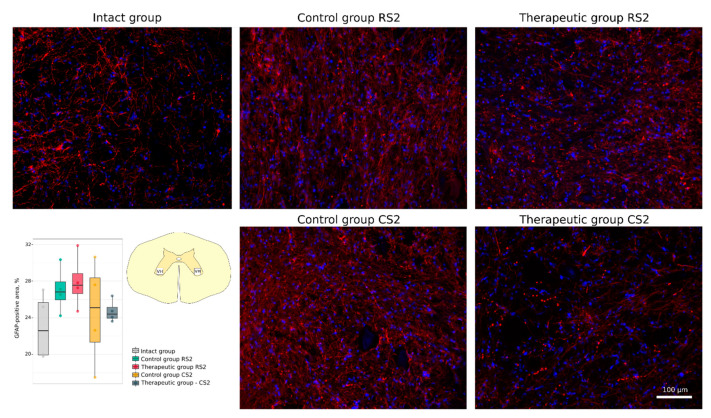
Immunofluorescence staining of the ventral horns of the spinal cords in the rostral (RS2) and caudal (CS2) segments 8 weeks after contusion injury with an antibody against glial fibrillary acidic protein (GFAP) (red). Nuclei were counterstained with DAPI (blue). Morphometric analysis demonstrates the mean (%) of the GFAP-positive area in the intact, control and therapeutic groups in the corresponding areas. The squares inserted in the schematic transverse spinal cord image demonstrates the areas used for immunofluorescence analysis, VH—ventral horn.

**Figure 12:**
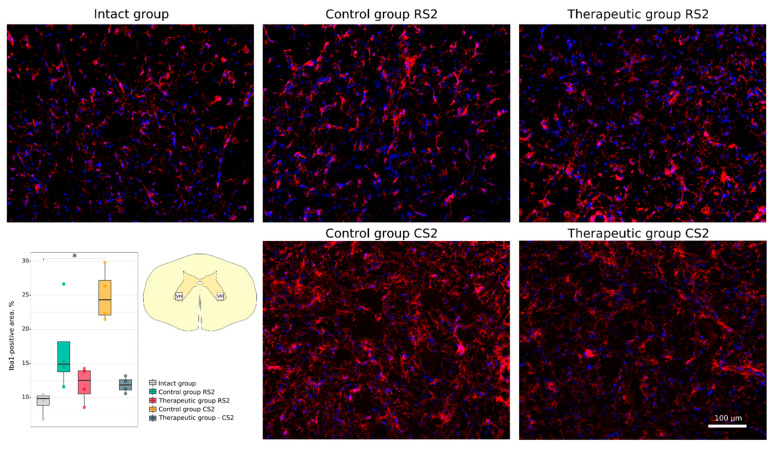
Immunofluorescence staining of the ventral horns of the spinal cords in the rostral (RS2) and caudal (CS2) segments 8 weeks after contusion injury with an antibody against ionized calcium binding adaptor molecule 1 (Iba1) (red). Nuclei were counterstained with DAPI (blue). Morphometric analysis demonstrates the mean (%) of the Iba1-positive area in the intact, control and therapeutic groups in the corresponding areas, * *p* < 0.05. The squares inserted in the schematic transverse spinal cord image demonstrates the areas used for immunofluorescence analysis, VH—ventral horn.

**Figure 13:**
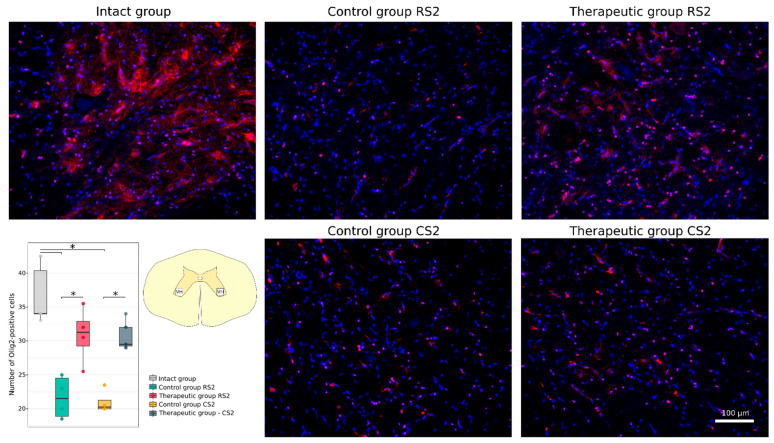
Immunofluorescence staining of the ventral horns of the spinal cords in the rostral (RS2) and caudal (CS2) segments 8 weeks after contusion injury with an antibody against oligodendrocyte transcription factor (Olig2) (red). Nuclei were counterstained with DAPI (blue). Morphometric analysis demonstrates the number of Olig2-positive nuclei in the intact, control and therapeutic groups in the corresponding areas, * *p* < 0.05. The squares inserted in the schematic transverse spinal cord image demonstrates the areas used for immunofluorescence analysis, VH—ventral horn.

Neuroglial cell remodelling was investigated using Abs to glial fibrillary acidic protein (GFAP) for astrocytes, to oligodendrocyte transcription factor (Olig2) for oligodendroglial cells, and to ionised calcium binding adaptor molecule 1 (Iba1) for microglia. No significant difference was found in the GFAP-positive areas in the rostral and caudal segments in the control (26.82 [25.96–27.91]; 25.11 [21.35–28.34]) and in the therapeutic (27.54 [26.63–28.83]; 24.39 [23.95–25.14]) groups relative to the intact group (22.59 [19.94–25.66]) (Figure 11). A significant increase in the Iba1-positive areas was observed in the caudal segment of the control group (24.31 [22.10–27.19]) when compared to the intact group (9.87 [8.87–10.26]) (*p* < 0.05) (Figure 12). At the same time, in the rostral segment of the control group (14.92 [13.77–18.17]), and in the rostral (12.55 [10.58–13.97]) and in the caudal (11.86 [11.12–12.63]) segments of the therapeutic group the Iba1-positive areas was not different from the intact group. The number of Olig2-positive cells in the rostral and caudal segments in the control group (21.5 [18.9–24.5]; 20.2 [20.0–21.2]) was significantly decreased in comparison to the intact group (34.0 [34.0–40.4]) (*p* < 0.05) and was as much lower than in the corresponding segments in the therapeutic group (31.2 [29.2–32.9]; 29.5 [29.2–32.0]) (*p* < 0.05) (Figure 13).

#### 3.4.2. Synaptic Gene Expression Analysis

**Figure 14:**
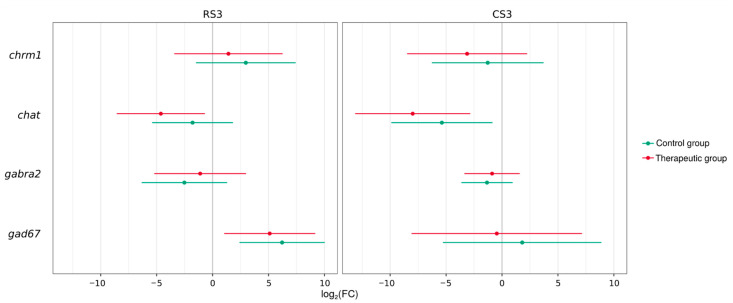
Synaptic gene expression in the rostral (RS3) and caudal (CS3) segments of the spinal cords 8 weeks after contusion injury. Analysis of the excitatory cholinergic system (pre-synaptic gene [*chat*] and the post-synaptic gene [*chrm1*] and inhibitory GABAergic system (pre-synaptic gene [*gad67*] and post-synaptic gene [*gabra2*]) is presented. The results are shown as an average log2(fold change) in control (green lines) and treated (pink lines) mini-pigs relative to the expression level in the intact group. Threshold cycles (Ct) were obtained for four animals (two repeats per animal) in each group and normalized by corresponding *gapdh* values.

Analysis of RT-PCR data on the neurotransmission gene expression demonstrated different patterns in the excitatory (cholinergic) and inhibitory (GABAergic) systems in the rostral (RS3) and caudal (CS3) segments in experimental and intact animals (Figure 14). Importantly, no significant differences in the average log2(fold change) was observed between control and therapeutic groups. They demonstrated similar changes in comparison with the intact group. The cholinergic system demonstrated a decrease in pre-synaptic gene expression (*chat*) in the rostral segment in the control and treated animals (0.024 [0.001–0.551]; 0.004 [0.000–0.140]) (*p* < 0.05) and in the caudal segment in the treated animals (0.040 [0.003–0.622]) (*p* < 0.05). The expression of the post-synaptic gene (*chrm1*) was not significantly changed in the control and treated animals in the rostral (0.412 [0.013–13.081]; 0.115 [0.003–4.746]) and in the caudal (7.833 [0.357–171.737]; 2.668 [0.093–76.812]) segments. GABAergic post-synaptic gene expression (*gabra2*) in control and treated animals was not significantly downregulated in both the rostral (0.395 [0.081–1.930]; 0.541 [0.097–3.003]) and caudal (0.174 [0.012–2.452]; 0.463 [0.027–7.965]) segments. However, the expression of the pre-synaptic gene (*gad67*) was upregulated in the control and therapeutic groups in the caudal (74.225 [5.302–1039.152]; 34.488 [2.046–581.474]) segment (*p* < 0.05) but was not changed in the rostral segment (3.492 [0.026–473.324]; 0.723 [0.004–142.272]).

## 4. Discussion

The natural limitations of regeneration in the central nervous system (CNS) is a key question that has to be addressed for the development of effective therapies for SCI. Moreover, the new regenerative therapies demonstrating the positive results in rodent models cannot be directly translated to neurorehabilitation therapy in humans with SCI. To facilitate this translation to the clinical trials experimental studies are supposed to be performed on large animals closer to humans on a morpho-physiological scale [39]. Herein we selected a mini-pig model with close to human spinal cord functional neuroanatomy, physiological, and biochemical characteristics [40,41].

The delivery of therapeutic genes encoding neurotrophic factors to the spinal cord after traumatic injury is a promising strategy that aims to increase the viability of neurons and prevent cell death, while stimulating axonal growth, remyelination, and restoration of lost neural connectivity. Meanwhile, electrical stimulation at different levels of the nervous system (various brain regions, intraspinal and epidural stimulation, ventral and dorsal root stimulation, and peripheral nerve stimulation) as well as skeletal muscle stimulation has become mainstream in experimental studies and clinical trials. Following our earlier observation that EES applied below the injury could facilitate movement in animals with complete SCI [36,37,38,42,43,44,45] and help subjects with clinically motor complete SCI restore volitional control below the injury [4,46], and taking into consideration the considerable evidence showing that long supraspinal tracts form synaptic connections above the injury with extensive reorganisation after SCI, we used the most advanced approach to coordinate restoration after SCI by targeting both sublesional and supralesional components of the translesional network [35]. Previously, we demonstrated the efficacy of translesional EES in mini-pigs with SCI in the recovery of motor function [28]. In the current study, we introduced an intravenous infusion of genetically-enriched autologous leucoconcentrate as a complementary therapy for multisite EES. SCI is accompanied with the destruction of the blood-brain barrier (BBB), which leads to leakage and increased homing of WBC that drive the inflammatory process [47]. Importantly, the gene modified WBC, after autoinfusion of the genetically enriched leucoconcentrate, had a high chance of reaching the injury epicentre and locally producing recombinant neurotrophic factors, as we demonstrated previously [32]. Moreover, under these conditions, the therapeutic molecules secreted by the gene modified WBC into the bloodstream may simply diffuse into the spinal cord tissue. Accordingly, intravenous infusion of the genetically enriched leucoconcentrate after neurotrauma proposes the delivery of recombinant proteins into the post-traumatic spinal cord and the modulation of neuroplasticity by recombinant VEGF, GDNF, and NCAM via direct action on neural and glial cells.

The general principles of the neuroontogenesis could be effectively employed in the development of the novel approaches for stimulation of neuroregeneration and neuromodulation. Several fundamental works demonstrated the redeployment of the embryonic gene regulatory network during regeneration [48,49], which suggests using the genes controlling neurogenesis as a powerful tool for stimulating neuroregeneration. In neuroontogenesis, VEGF signaling is involved in axon guidance and synaptogenesis [50]. GDNF has a pronounced neuroprotective effect and stimulates axon growth and synapse formation in the developing nervous system [51]. NCAM as well plays a critical role in neurogenesis, its expression on the neurons and glial cells membrane modulate the interaction of the cells with extracellular matrix proteins and thus migration of neurons and navigation of neurites growth during synaptogenesis [52]. These data together with our results on the positive effect of the autologous genetically-enriched leucoconcentrate confirm our hypothesis on rationality to use recombinant genes, encoding VEGF, GDNF, and NCAM for stimulation neuroregeneration in CNS.

Complex analysis of multisite translesional EES in combination with intravenous infusion of genetically-enriched autologous leucoconcentrate on the recovery of mini-pigs with SCI, when compared with control animals, demonstrated: (1) improvements in locomotor activity (better behavioural and joint kinematics performance, restoration of electromyography characteristics); (2) the increased survivability of post-traumatic spinal cord cells (lower number of caspase-3-positive cells and a larger immunopositive area for Hsp27 and KCC2 in the rostral and caudal segments); (3) positive reorganisation of glial cells (higher number of myelin-forming Olig2-positive cells and a lower number of microglial Iba1-positive cells) in the rostral and caudal segments; (4) restoration of neural connections (larger immunopositive area for synaptophysin, PSD95, and βIII-tubulin in the caudal segment). Altogether, these observations suggest that translesional EES combined with infusion of autologous genetically-enriched leucoconcentrate had a positive therapeutic effect on restoration after SCI in mini-pigs.

The therapy of hereditary diseases of the immune system, based on the correction of a mutant gene by delivering a functional gene into the patient’s leukocytes, is the first method of gene replacement therapy that was successfully implemented in clinical practice [53]. The use of WBC from the patient’s peripheral blood as cellular carriers of recombinant genes for the temporary production of therapeutic molecules to correct a specific pathological process appears to be a new approach in gene therapy. To date, there are no ready-made, tested, and effective cell preparations containing genetic material for the temporary production of biologically active protein molecules. Accordingly, we have developed a simple, safe, and economically efficient method to prepare an autologous leucoconcentrate, enriched with recombinant genetic material for personalised precision gene therapy.

The rationale for using WBC compared to other cell-mediated therapies is also based on the following benefits: (a) WBC possess high synthetic and secretory activity; (b) WBC are able to effectively migrate from the bloodstream to any body tissue; (c) CD46 on the WBC surface provides efficient transduction with chimeric adenoviral vectors (Ad5/35F); (d) WBC can be transduced with one or more recombinant therapeutic genes; (d) ex vivo WBC transduction prevents the direct toxic and immunogenic effects of the adenoviral vector in the recipient; (f) genetically-enriched leucoconcentrate provides temporary production of recombinant biologically active protein molecules in the recipient; (g) the concentration of genetic vector used for the transduction of WBC or the amount of genetically-enriched leucoconcentrate used for infusion allows researchers/clinicians to control the level of therapeutic molecule production by WBC in the recipient; (h) genetically-enriched leucoconcentrate can be infused systemically (intravenously) or locally (intramuscularly and subcutaneously); (i) repeated autoinfusion of genetically-enriched leucoconcentrate is possible; (j) blood centres can produce genetically-enriched leucoconcentrate using approved genetic constructs that contain human therapeutic genes.

**Figure 15:**
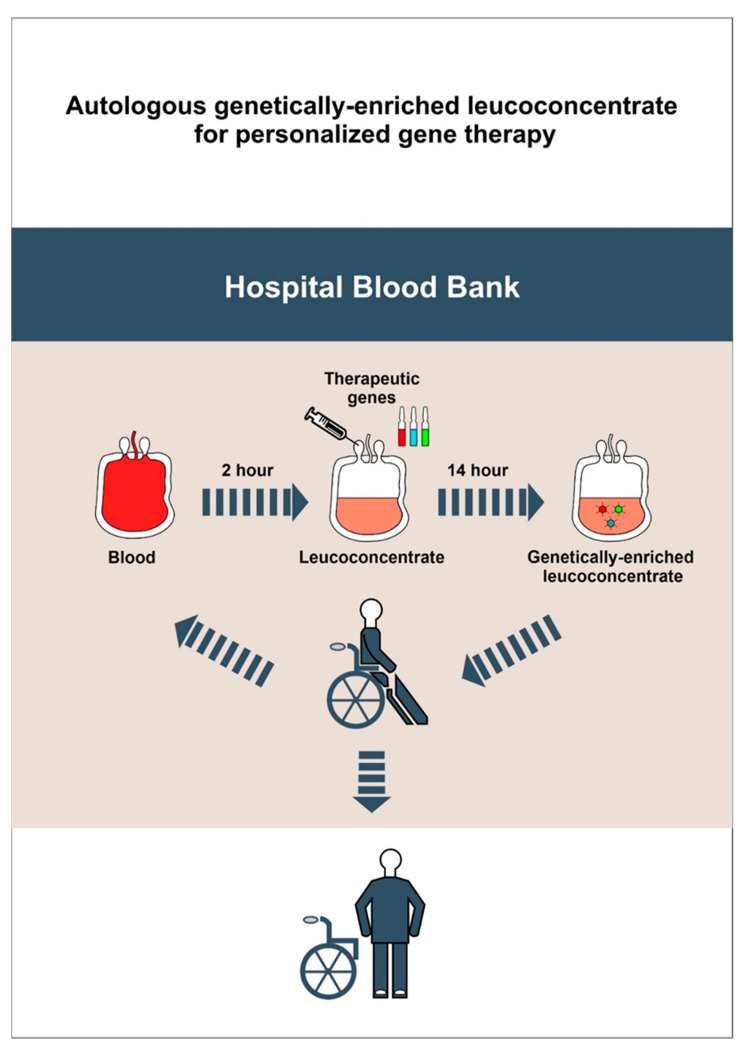
A simple, safe and economic approach for personalized precision *ex vivo* gene therapy based on autoinfusion of the genetically-enriched leucoconcentrate prepared from a routine unit of patient peripheral blood.

An autologous leucoconcentrate enriched with cDNA encoding human vascular endothelial growth factor 165 (VEGF165), human glial cell line-derived neurotrophic factor (GDNF), and human neural cell adhesion molecule 1 (NCAM1) demonstrated efficacy as complementary gene therapy with EES for SCI treatment in a mini-pig model. The technology developed and tested in this study can be used for other diseases, such as ischemic stroke, amyotrophic lateral sclerosis, and Alzheimer’s disease. At the same time, the use of an autologous leucoconcentrate, enriched with genetic material, is not limited to stimulating neuroregeneration. Depending on the therapeutic gene or combination of genes in the composition of the leucoconcentrate, it can be used for the enhancement of pathogenic therapy for other somatic or infectious diseases and used as a complementary therapy, for example, in the bypass grafting of blood vessels co-treated with VEGF gene therapy, osteosynthesis of bones co-treated with bone morphogenic protein (BMP) gene therapy, antimicrobial interventions for purulent diseases combined with lactoferrin gene therapy (Figure 15). Moreover, it may be possible to freeze and store autologous leucoconcentrate enriched with genetic material prior to using it for autoinfusion, if necessary, in persons with an increased health risk (military, firefighters, athletes, etc.).

This study aimed to combine two different approaches (neuromodulation and ex vivo gene therapy) to control a variety of pathogenic mechanisms of neurodegeneration and stimulate neuroregeneration after SCI. For the first time in a mini-pig SCI model, neuromodulation with supra- and sub-lesional EES accompanied with training on a treadmill was enhanced with ex vivo gene therapy based on intravenous infusion of an autologous leucoconcentrate enriched with *vegf165*, *gdnf*, and *ncam1*. Behavioural, electrophysiological, and histological studies revealed the therapeutic efficacy of the combined electro- and gene therapies on the post-traumatic spinal cord recovery in mini-pigs.

## Figures and Tables

**Table 1:** Nucleotide sequences of the primers and products of real time PCR.

Genes and Nucleotide Sequences	Product Length, bp	GC%	T_m_, °C
*vegf165* F: CTTGCTCTATCTTTCTTTGG R: GCTGCTCTACCTCCACCATG	400	40.00	45
		60.00	45
*gdnf* F: GGATGTCGTGGCTGTCTGCCTGG R: TGCCCCTCTGGCCTCTCCGACC	300	65.20	55
		72.70	55
*ncam1* F: GCCGTGATTGTGTGTGATGT R: GCCTCGTCGTTCTTATCCAC	450	50.00	45
		55.00	45
*chat* (NM_001001541) F: CCATGCCGGATTCGGAGAA R: CCCATTGGGTACCACAGGAC	263	57.89	59.85
		60.00	60.03
*chrm1* (NM_214034) F: GAAAAGCTTGGCTCAGAGGGA R: ATGACATAGTGGGACCGTCG	260	52.38	60.27
		55.00	59.26
*gad67* (D31849) F: GCCGACCTCATTGTCCGTAT R: GTGTTCTCCACCCCACACAA	184	55.00	59.90
		55.00	60.11
*gabra2* (XM_013978645) F: CACGCCAGAACCCAACAAGA R: GTACATGGCAAAACAAACCAGG	244	55.00	60.82
		45.45	58.61
*gapdh* (NM_001206359) F: CCGTGTGTTCCGTGCATTG R: TGCCGTGGGTGGAATCATAC	198	57.89	60.08
		55.00	60.11

GC%: GC content, Tm: melting temperature.

**Table 2:** Antibodies used in immunofluorescent analysis.

Antibody Against:	Host	Dilution	Source
Beta III Tubulin	Rabbit	1:200	Abcam (Cat # ab18207)
Caspase 3	Rabbit	1:200	Cell Signaling Technology (Cat # 9661)
Glial cell-line derived neurotrophic factor (GDNF)	Rabbit	1:100	Santa Cruz (Cat # sc-9010)
Glial fibrillary acidic protein (GFAP)	Mouse	1:200	Santa Cruz (Cat # sc-33673)
Ionized calcium binding adaptor molecule 1 (Iba1)	Rabbit	1:150	Abcam (Cat # ab178847)
The K^+^–Cl^−^ cotransporter isoform 2 (KCC2)	Rabbit	1:200	Abcam (Cat # ab49917)
Heat shock protein 27 kDa (Hsp27)	Rabbit	1:200	Abcam (Cat # ab12351)
Oligodendrocyte transcription factor 2 (Olig2)	Rabbit	1:100	Abcam (Cat # ab18258)
Neural cell adhesion molecule (NCAM)	Rabbit	1:100	Abcam (Cat # ab9272)
Postsynaptic density protein 95 kDa (PSD95)	Rabbit	1:200	Abcam (Cat # ab18258)
Synaptophysin	Rabbit	1:100	Abcam (Cat # ab32127)
Vascular endothelial growth factor (VEGF)	Rabbit	1:300	Santa Cruz (Cat # sc-152)
Mouse IgG conjugated with Alexa 488	Donkey	1:200	Invitrogen (Cat # A-21202)
Rabbit IgG conjugated with Alexa 488	Donkey	1:200	Invitrogen (Cat # A-21206)
Rabbit IgG conjugated with Alexa 647	Donkey	1:200	Invitrogen (Cat # A-31573)

**Table 3:** Complete blood count for venous blood and genetically-enriched leucoconcentrate.

Sample	WBC (10⁹/L)	RBC (10^12^/L)	PLT (10⁹/L)	LYM (10⁹/L)	MON (10⁹/L)	GRAN (10⁹/L)	Volume (mL)
VB	13.2 ± 3.3	6.5 ± 0.9	710.7 ± 88.8	7.0 ± 1.5	0.6 ± 0.3	5.6 ± 1.6	50.0 ± 0.0
GEL	8.0 ± 3.7	0.2 ± 0.2	196.0 ± 155.2	2.9 ± 2.5	0.6 ± 0.4	4.5 ± 3.9	49.3 ± 14.4
P	0.0469	0.0156	0.0156	0.0156	1	0.3750	0.9325

VB: venous blood; GEL: genetically-enriched leucoconcentrate; WBC: white blood cell; RBC: red blood cell; PLT: platelet; LYM: lymphocytes; MON: monocytes; GRAN: granulocytes. Results are presented as the mean ± standard deviation.

## Data Availability

The data presented in this study are available on request from the corresponding author.
